# Efficacy of manual acupuncture versus placebo acupuncture for generalized anxiety disorder (GAD) in perimenopause women: study protocol for a randomized controlled trial

**DOI:** 10.1186/s13063-021-05756-x

**Published:** 2021-11-24

**Authors:** Xin Liu, Xiaoyan Xie, Yingjia Li, Meichen Li, Yuting Wang, Nanbu Wang, Lixing Zhuang, Muxi Liao

**Affiliations:** 1 Clinical Medical College of Acupuncture Moxibustion and Rehabilitation, Guangzhou University of Chinese Medicine, Guangzhou, 510000 China; 2 The Second Clinical Medical College, Guangzhou University of Chinese Medicine, Guangzhou, 510006 China; 3 Guangdong Provincial Hospital of Chinese Medicine, Guangzhou, 510120 Guangdong China; 4 Department of Rehabilitation, The First Affiliated Hospital of Guangzhou University of Chinese Medicine, Guangzhou, 510000 China

**Keywords:** Generalized anxiety disorder, Acupuncture, Protocol

## Abstract

**Background:**

Generalized anxiety disorder (GAD) is common among perimenopausal women. Acupuncture may be an effective treatment for GAD, but evidence is limited. The pathogenesis of GAD is not yet clear, but it is related to the hypothalamic-pituitary-adrenal axis and its excretion, cortisol (CORT), and adrenocorticotropic hormone (ACTH). The object of this study is to evaluate the efficacy of manual acupuncture (MA) versus placebo acupuncture (PA) for perimenopausal women with GAD.

**Methods:**

This study is a single-center, randomized, single-blind clinical trial that will be conducted in the First Affiliated Hospital of Guangzhou University of Chinese Medicine. A total of 112 eligible GAD patients will be randomly assigned (1:1) to receive MA (*n*=56) or PA (*n*=56) three times per week for 4 weeks. The primary outcome measure will be the HAMA score. The secondary outcome measures will be the GAD-7 and PSQI scores and the levels of CORT and ACTH. The evaluation will be executed at baseline, 2 weeks, the end of the treatment, and a follow-up 3-month period. All main analyses will be carried out based on the intention-to-treat (ITT) principle.

**Discussion:**

This study intends to compare the efficacy between MA and PA in the treatment of perimenopausal women with GAD and to further study the mechanisms underlying the effect.

**Trial registration:**

Chinese Clinical Trial Registry ChiCTR2100046604. Registered on 22 May 2021.

## Background

Generalized anxiety disorder (GAD) is a kind of chronic mental disorder that includes both mental and physical symptoms [1]. GAD patients have general apprehensiveness or worry that is not restricted to any particular stimulus as the primary clinical feature [2]. According to epidemic research in Europe, the incidence rate of GAD is 6.2% throughout the lifetime [3]. Moreover, females have 1.5 to 2 times more exposure to GAD than males [4]. To our knowledge, the prevalence of females aged between 45 years old and 54 years old is the highest [5]. This phenomenon indicates that perimenopausal women are more likely to suffer from GAD. As one of the first-line pharmacotherapies for GAD [6], serotonin reuptake inhibitors (SSRIs) inhibit the reuptake of serotonin to increase the concentration of serotonin in the synaptic cleft to improve the level of serotonin in the brain which is believed to attenuate anxious symptoms [7]. However, patients’ conditions might be aggravated in the first week when first taking SSRIs [8]. Therefore, some patients refuse to take drugs because of adverse events. They seek for the help of psychiatrists and some of them probably receive cognitive behavioral therapy (CBT). Studies [9, 10] have shown that CBT is an effective treatment for GAD. However, due to the shortage of professionals and high cost, CBT is not beneficial for most patients in China [11]. Therefore, patients turn to find help from alternative therapies such as acupuncture.

Although the pathogenesis of GAD is not yet clear, current studies believe that it is related to abnormal secretory function of the hypothalamic-pituitary-adrenal axis (HPA axis) [12]. Cortisol (CORT) synthesized by the adrenal cortex is one of the final products of the HPA axis. CORT and adrenocorticotropic hormone (ACTH) levels in GAD patients are higher than those in normal people [13, 14]. The elevation of CORT and ACTH is detected as negative feedback by the hypothalamus which downregulates the stress response [15, 16]. Therefore, the decline in CORT and ACTH levels could predict the degree of improvement that a treatment produces [17].

Therefore, we designed a randomized controlled trial that focus on the efficacy of manual acupuncture (MA) versus placebo acupuncture (PA) on GAD in perimenopausal women. To evaluate the therapeutic efficacy of GAD, the primary outcome measure used in our protocol is the change in scores on the Hamilton Anxiety Scale (HAMA). The secondary outcome measures are the changes in scores on the Generalized Anxiety Disorder Scale (GAD-7) and Pittsburgh Sleep Quality Index (PQSI), and laboratory indicators related to the current possible pathogenesis of GAD, mainly including CORT and ACTH. This protocol aims to explore the efficacy of MA in GAD patients who would not prefer drug or CBT therapies. Meanwhile, we hope to explore the possible mechanism of acupuncture on GAD by measuring the levels of CORT and ACTH in serum.

## Methods

### Study design

**Fig. 1:**
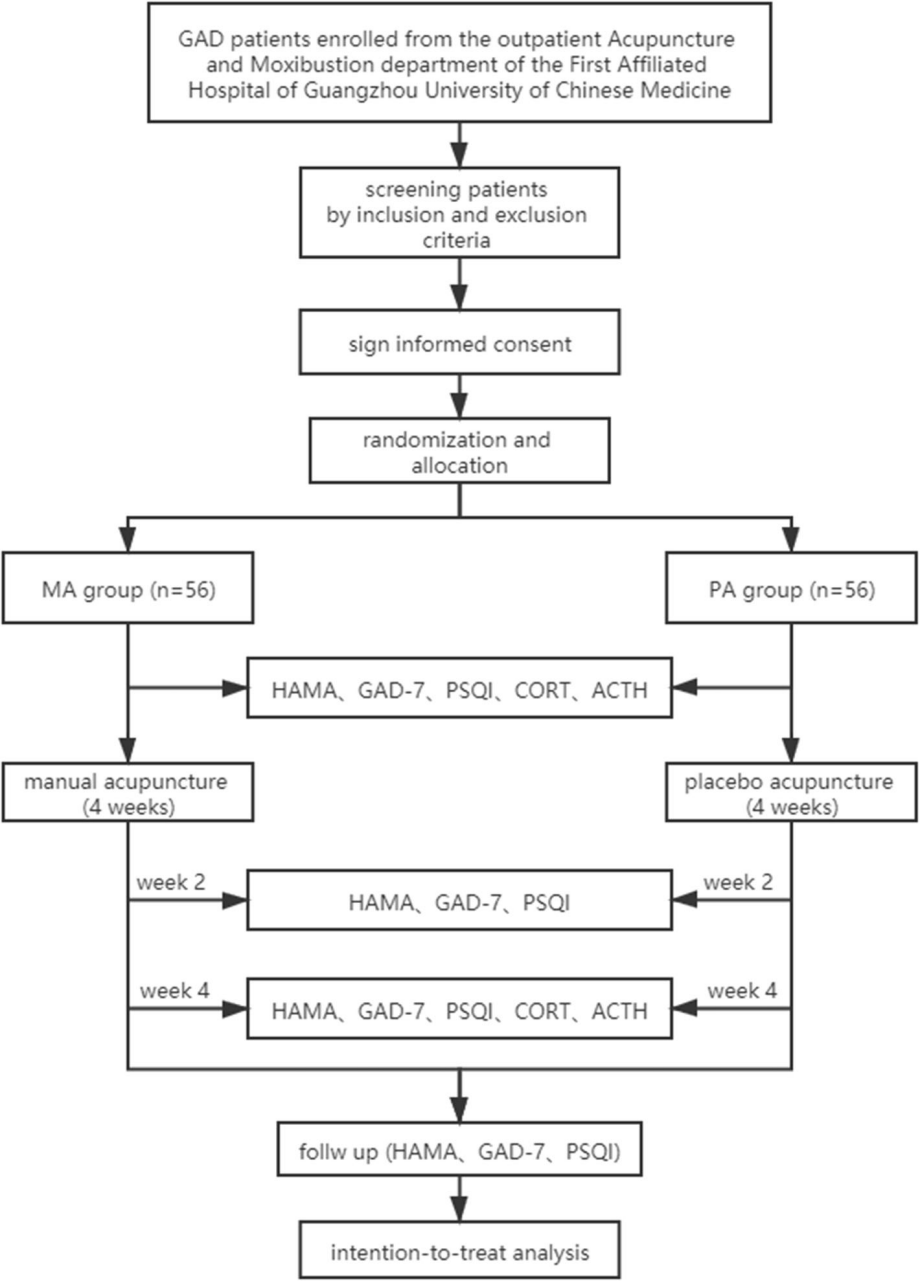
Flow chart

This is a randomized controlled and patient-and-assessor-blind trial. A total of 112 participants will be enrolled from the outpatient Acupuncture and Moxibustion Department of the First Affiliated Hospital of Guangzhou University of Chinese Medicine. The flow chart is shown in Fig. 1. The protocol is composed based on the SPIRIT checklist [18] and the Declaration of Helsinki [19]. The trial was approved by the Ethics Committee of the First Affiliated Hospital of Guangzhou University of Chinese Medicine (K[2021]014) and has been registered at the Chinese Clinical Registry (ChiCTR2100046604).

### Sample size

According to preliminary trials [20, 21], we chose the HAMA score as the outcome to calculate the sample size. After treatment, the HAMA score of MA was 14.0±3.2, and the HAMA score of PA was 19.4±5.3. PASS version 15.0 (NCSS, LLC. Kaysville, UT, USA) was used to estimate the sample size with a power level of 90% and a two-sided significance level of 5%. After calculation, we set the sample size of 44 patients in each group to observe the significant difference between the two groups. With a 20% withdrawal rate, we plan to enroll a total of 112 patients with 56 patients in each group.

### Randomization and allocation concealment

An independent researcher sets a random number as a seed and used IBM SPSS Statistics version 26 (IBM SPSS Inc., Chicago, USA) to generate a random allocation sequence. According to the size of the random number sequence, patients who received the first 56 random numbers will be allocated into the MA group, and patients who received the last 56 random numbers will be allocated into the PA group in a 1:1 ratio. The researcher will put the cards with written random numbers and group assignments into sealed envelopes. The intervenors will allocate enrolled patients into the MA group or PA group on the basis of the card in the envelope.

### Blinding

**Fig. 2:**
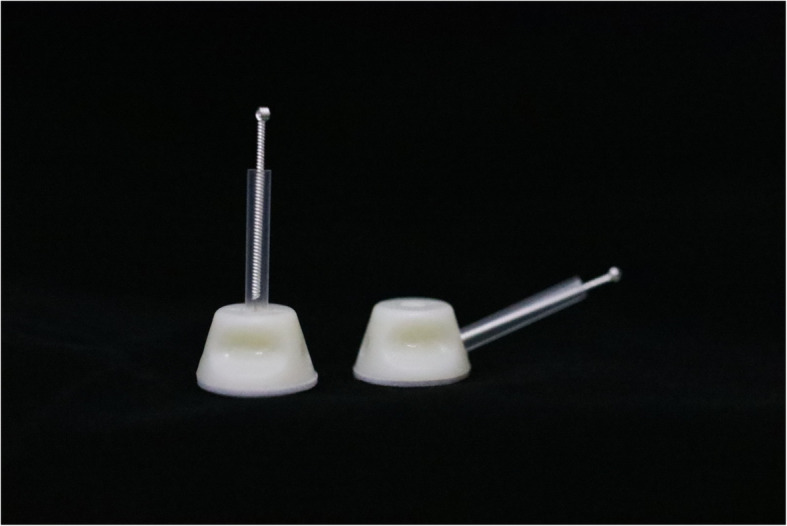
Different needling angles

**Fig. 3:**
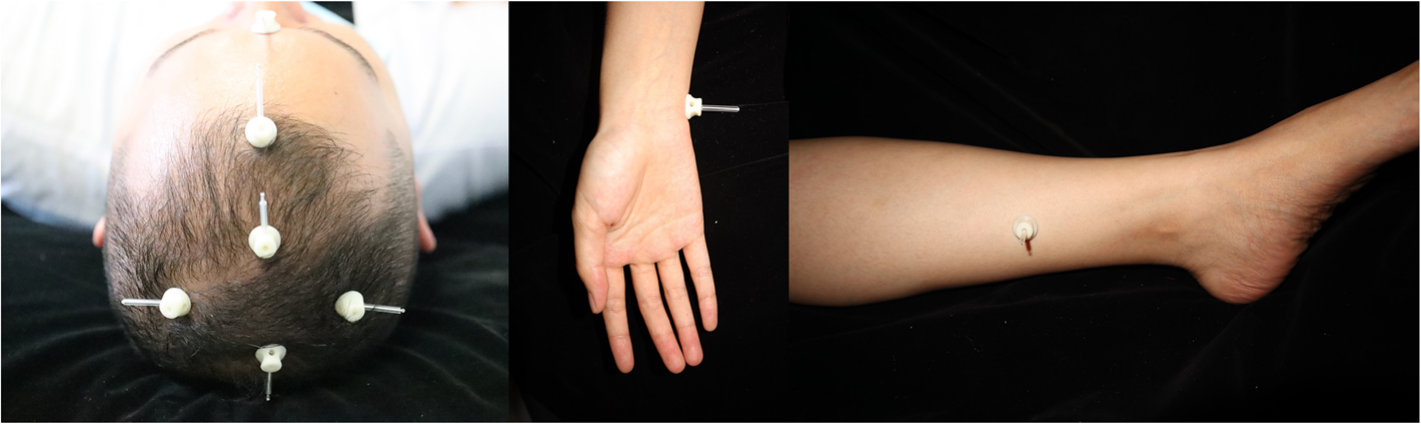
Head and body

Due to the nature of acupuncture, it is difficult to blind patients and intervenors at the same time. We designed a new placebo acupuncture needle pedestal to blind patients, and the pedestal was applied for a patent in China (202121352221.7). The new placebo needle can be used at different positions with the acupoints and at different needling angles (Fig. 2). The PA group used a special needle (φ0.30 × 40 mm, produced by Huanqiu Co. Ltd., China) in which the tip is blunt so that the needle could not be pricked into the skin. In addition, the diameter of the blunt needle is 0.3 mm. It is thin enough to make patients feel like they are pricked. The pedestal is opaque so patients would not know whether the needle is pricked. Therefore, patients are unaware of whether they receive MA or PA. In addition, there is adhesive tape below the base to ensure that the whole pedestal can adhere to the head and body (Fig. 3). To test the participant-blinding effects, the first two patients will be randomly selected from the study to guess whether they have received MA or PA within 5 minutes after one of the treatment sessions in weeks 2 and 4. In addition, researchers who analyze the data are unaware of group assignments either to minimize the potential source of bias.

### Patient recruitment

A total of 112 GAD patients will be recruited through, but not limited to, posters pasted in the hospital lobby and social media (WeChat), which can be seen by potential patients. Meanwhile, we will cooperate with the gynecology and psychology departments of the hospital for potential patients to ensure the target sample size is reached. Eligible patients who meet the inclusion criteria and exclusion criteria will be enrolled in the outpatient department of the First Affiliated Hospital of Guangzhou University of Chinese Medicine.

### Inclusion criteria

Patients who meet all of the following criteria will be allowed to enrollment:

1. Aged 45 to 55 years old (female only).
2. Meet the diagnostic criteria of DSM-5 [1] for GAD.
3. Excessive anxiety and worry (apprehensive expectation), occurring more days than not for at least 6 months.
4. The individual finds it difficult to control the worry.
5. The anxiety and worry are associated with three (or more) of the following six symptoms (with at least some symptoms having been present for more days than not for the past 6 months):

- Restlessness or feeling keyed up or on edge.
- Being easily fatigued.
- Difficulty concentrating or mind going blank.
- Irritability.
- Muscle tension.
- Sleep disturbance (difficulty falling or staying asleep, or restless, unsatisfying sleep).

(4) The anxiety, worry, or physical symptoms cause clinically significant distress or impairment in social, occupational, or other important areas of functioning.
(5) The disturbance is not attributable to the physiological effects of a substance (e.g., a drug of abuse, a medication) or another medical condition (e.g., hyperthyroidism).
(6) The disturbance is not better explained by another mental disorder (e.g., anxiety or worry about having panic attacks in panic disorder).
(7) The score of HAMA is between 14 and 28.
(8) No anti-anxiety drugs or other psychotropic drugs in the last 2 weeks.
(9) No participation in any other research in the last 1 month.

### Exclusion criteria

Patients who meet any of the following criteria will be excluded:

1. Diagnosed by other severe illnesses such as heart failure, tumors, renal failure, and so on.
2. Diagnosed by psychotic illness such as schizophrenia, agoraphobia, and so on clearly.
3. Addicted to alcohol or drugs in the past 3 months.
4. Afraid of acupuncture.
5. Pregnancy or breastfeeding.

### Drop out criteria

Patients who meet any of the following criteria will be excluded from the study:

1. Cannot complete all treatment sessions.
2. Outbreaks of serious illness threatening life include disability, cancer, organ failure, and so on.
3. Unwilling to comply with acupuncture treatment.
4. Anti-anxiety drugs or other psychotropic drugs were administered when enrolled in the study.
5. Pregnancy when enrolled in the study.
6. Patient quits the study treatment by herself.

### Inventions

**Fig. 4:**
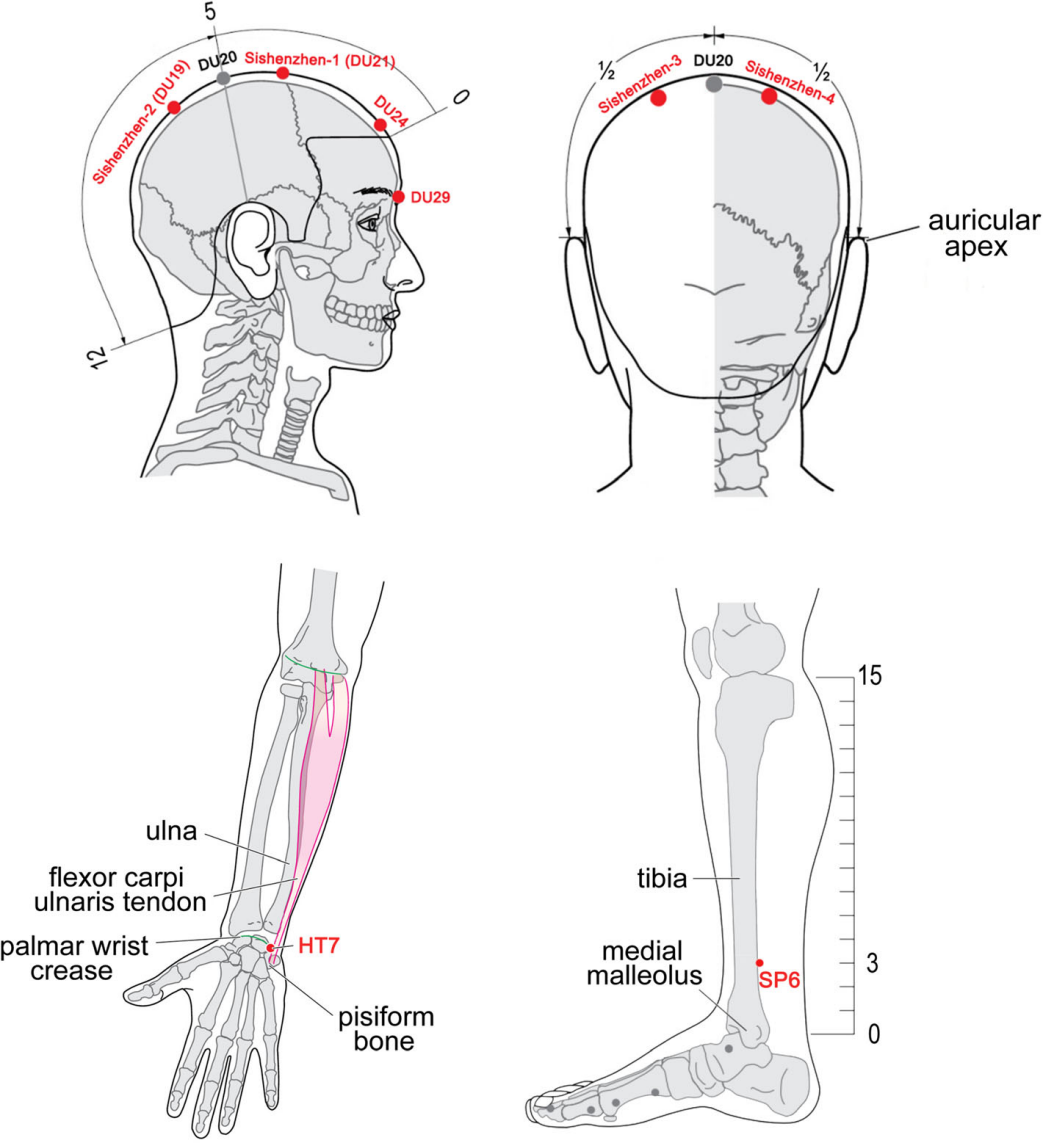
Acupoint locations

All eligible patients will receive 12 sessions of MA or PA (4 weeks, three times a week). Each session will take 30 min. The acupuncture therapists have acupuncture education from Guangzhou University of Chinese Medicine (range 1–3 years of full-time academic studies) or have clinical experience in the First Affiliated Hospital of Guangzhou University of Chinese Medicine (range 2–5 years). In addition, all intervenors are trained to completely understand the standard of operation. The acupoints of the MA and PA groups are the same, including Sishenzhen (one of the main acupoints in Jin’s 3 Needle [22]), Shenting (DU24), Yintang (DU29), Shenmen (HT7), and Sanyinjiao (SP6). The acupoint locations are according to the “2006 People’s Republic of China National Standard” (GB/T1234-2006) with the exhibition in Fig. 4. The acupuncture operation will be performed according to the textbook of the 10th Five-Year Plan of the Ministry of Health. Enrolled patients will be treated one-on-one by a trained acupuncturist from beginning to the end. In addition, an independent assistant will follow the intervention procedures to ensure the consistency of treatment.

### MA group

Patients in the MA group will be pricked by disposable, stainless steel needles (φ0.30 × 40 mm, produced by Huanqiu Co. Ltd., China) through our device. The needles will be inserted into the skin to ensure that patients have a Deqi sensation [23] which is considered the essential reason for the efficacy of acupuncture [24]. Depth of puncture is approximately 25–30 mm. The needles will be manipulated three times (at the start, middle, and end of every session) by twirling and lifting until the Deqi sensation occurs.

### Placebo acupuncture (PA) group

Patients in the PA group will receive placebo acupuncture in which the needles will not be inserted into the skin and will not induce the Deqi sensation. The blunt-tipped placebo needle (φ0.30 × 40 mm, produced by Huanqiu Co. Ltd., China) can provide participant-blinding effects with a similar appearance to conventional needles but no skin penetration.

### Outcome measure

**Fig. 5:**
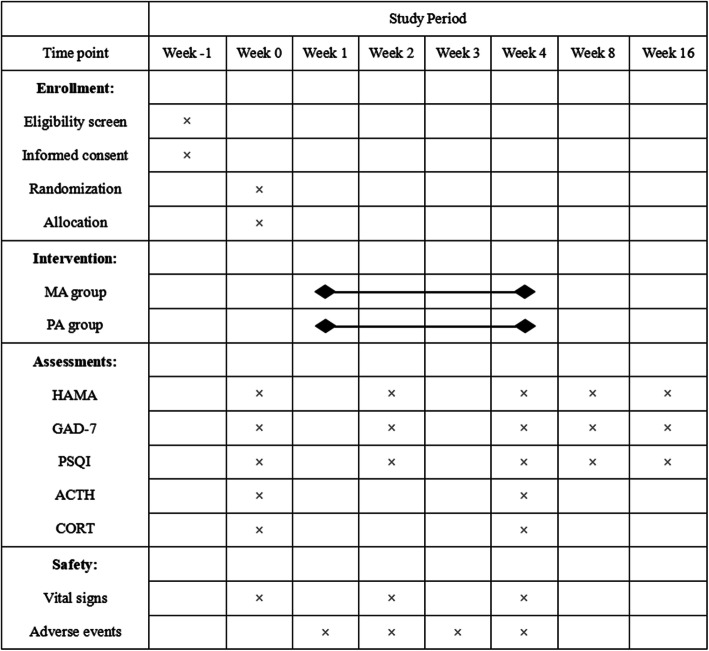
The schedule of the whole procedure

The schedule of the whole procedure is shown in Fig. 5. An independent assessor who does not know the group assignment will collect outcome data for analysis.

### Primary outcome

#### HAMA score

The primary outcome of this study is the HAMA [25] score, which measures the severity of anxiety. The HAMA is a 14-item scale rated on a 4-point scale with a total score ranging from 0 to 56. A higher score means a higher range of anxiety. According to studies, a score higher than 28 indicates severe anxiety, a score between 28 and 21 indicates moderate anxiety, a score between 20 and 14 indicates mild anxiety, and a score lower than 14 indicates no anxiety. HAMA will be assessed on the first day, the 14th day, and the 28th day of the trial.

### Secondary outcome

#### GAD-7 score

The secondary outcome of this study is the GAD-7 [26] score, which measures the severity of GAD particularly. The GAD-7 is a 7-item scale rated on a 3-point scale with a total score ranging from 0 to 21. In addition, the GAD-7 score indicates the level of general anxiety. The higher the GAD-7 score is, the more anxious the patient is. If the patient is enrolled, GAD-7 will be assessed on the first day, the 14th day, and the 28th day of the trial.

#### PSQI score

The secondary outcome of this study is the PSQI [27] score, which measures the quality of sleep in the last month. The PSQI is a 19-item questionnaire, divided into 7 parts: sleep quality, sleep latency, sleep duration, habitual sleep efficiency, sleep disturbances, use of sleeping medication, and daytime dysfunction. The PSQI score is inversely proportional to the quality of sleep. PSQI will be assessed on the first day, the 14th day, and the 28th day of the trial.

#### CORT and ACTH

It has been suggested that anxious subjects demonstrate greater CORT and ACTH concentrations [28]. In this study, we will draw serum samples and investigate CORT and ACTH before and after acupuncture. An enzyme-linked immunosorbent assay (ELISA) will be used to measure CORT and ACTH. These related tests will be carried out by the clinical laboratory of the First Affiliated Hospital of Guangzhou University of Chinese Medicine.

### Safety outcome

Researchers will record body temperature, respiratory rate, pulse rate, and blood pressure on the first day, the 14th day, and the 28th day of the treatment. Adverse events (AEs) caused by acupuncture should be truthfully recorded in case report forms (CRFs). In addition, intervenors should treat patients with AEs as soon as possible to comfort patients and eliminate AEs. In the meanwhile, we will inform AEs to the Ethics Committee of the First Affiliated Hospital of Guangzhou University of Chinese Medicine. After treatment of AEs, researchers will ask patients whether they would like to continue the trial or not and respect their will.

### Ancillary and post-trial care

After the whole treatment, patients who received PA treatment will receive 12 sessions of MA treatment to compensate. If patients who receive MA treatment want to continue treatment, they can appoint and be treated in the outpatient department.

### Follow-up

After the completion of 12 sessions of acupuncture, we will follow patients via telephone or WeChat to score the HAMA, GAD-7, and PSQI scores in the first month and the third month to determine whether the efficacy can be maintained.

### Data monitoring and analysis

Data will be saved on paper as recorded by the assessors. All original data will be stored in the medical record room of the First Affiliated Hospital of Guangzhou University of Chinese Medicine for 3 years. An independent assistant will monitor the process of collecting data and the Ethics Committee of the First Affiliated Hospital of Guangzhou University of Chinese Medicine will carry out audits at regular intervals.

Two independent study assistants will input the data, check by themselves whether the input data have discrepancies, and correct them. IBM SPSS Statistics version 26 (IBM SPSS Inc., Chicago, USA) will be used to analyze the data. The HAMA, GAD-7, and PSQI scores assessed in each group will be subjected to normality tests. If the scores obey a normal distribution, a one-way analysis of variance will be performed. If not, the Kruskal-Wallis rank sum test will be performed. The repeatedly assessed HAMA, GAD-7, and PSQI scores in the MA or PA group will be subjected to a normality test. If the scores obey a normal distribution, repeated measurement analysis of variance will be performed. If not, the Friedman rank sum test will be performed. If the significance of the *F*-test of the univariate linear model is greater than 0.05, CORT and ACTH will be subjected to covariance analysis. If not, the Kruskal-Wallis rank sum test will be performed.

All statistical tests will be two-sided, and the significance level will be set at 0.05. If the patient drops out, we will follow the intention-to-treat (ITT) and use the last score or serological indices assessed as the final score or indices.

### Quality control

Two independent study assistants will input the data to avoid mistyping. Before the beginning of the trial, all intervenors will be trained, and another assistant will follow the intervention procedures and stay in the outpatient department to ensure consistency of acupuncture intervention among intervenors. In addition, quality inspectors will check the CRFs randomly to ensure that there are no recorded errors.

## Discussion

According to recent meta-analyses or systematic reviews [29, 30], acupuncture shows to have a better treatment efficacy for GAD, but the conclusions are limited. Many articles have compared efficacy between MA and PA, but the conclusions are different. Some reviews [31–34] have concluded that MA is definitely better than PA, and other reviews [35] found no difference between MA and PA. The jury is out on whether or not there is an existing acupuncture placebo effect. To the best of our knowledge, no trial has studied the efficacy of MA versus CBT in treating GAD. In the present research [36], CBT combined with electroacupuncture (EA) resulted in a lower HAMA score than CBT or EA alone. In China, CBT is not as widespread as in Western countries. The expense of CBT is much higher than that of acupuncture, so a few GAD patients will choose CBT to treat GAD. Therefore, this trial compared MA and PA to determine whether there is a placebo effect and provide clinical evidence of the placebo effect of acupuncture.

There are many types of anxiety disorders and GAD is one of the most common types. The HAMA, introduced in 1959, was widely used in evaluating anxiety levels in lots of clinical trials. The GAD-7, introduced in 2006, was designed for GAD; this is a special type of anxiety disorder. However, RCTs about anxiety used GAD-7 as an outcome are limited. Due to the specificity of the GAD-7 and the generalization of the HAMA, we will use the HAMA scores as the primary outcome and the GAD-7 scores as the secondary outcome to measure the change in patients’ anxiety levels. We will use the PSQI scores as a secondary outcome because there is a high rate of comorbidity between insomnia and GAD [37, 38]. Less sleeping will aggravate patients’ mental burden and worsen their anxiety. Therefore, we want to determine whether there is a connection between GAD and sleeping. In addition, we will collect serum samples of GAD patients to evaluate the changes in CORT and ACTH during treatment. If the levels of CORT and ACTH are associated with the level of GAD, acupuncture may treat GAD via the HPA axis.

This study has room for improvement. First, due to the nature of acupuncture, we spare no effort to maintain blinding, but we still cannot find a perfect way to achieve double blinding. We can make the pedestal of acupuncture needles that look the same and simulate the feeling of being needled by touching with semi blunt needles. Therefore, patients are unaware of whether they receive MA or PA. However, experienced acupuncturists can feel the difference in whether needles prick the skin and achieve Deqi or not. Second, this study is a single-center trial, and we hope that this study will provide more clinical evidence and design a multi-center trial to confirm the efficacy of acupuncture in treating GAD.

## Trial status

The trial is currently being carried out right now and the end date of enrollment is expected on May 1, 2022.
